# A multi-metric evaluation of a sustainable capillary electrophoresis method for simultaneous therapeutic drug monitoring of Nilotinib and Ofloxacin in oncology clinics

**DOI:** 10.1038/s41598-026-46953-5

**Published:** 2026-05-06

**Authors:** Weam M. Othman, Nourah Z. Alzoman, Ibrahim A. Darwish, Awadh M. Ali, Fatma F. Abdallah, Samah S. Saad, Nehal F. Farid

**Affiliations:** 1 Pharmaceutical Analytical Chemistry Department, Faculty of Pharmacy, Misr University for Science and Technology, 6th October City, Egypt; 2 Department of Pharmaceutical Chemistry, College of Pharmacy, King Saud University, P.O. Box 2457, Riyadh, 11451 Saudi Arabia; 3 Pharmaceutical Analytical Chemistry Department, Faculty of Pharmacy, Beni-Suef University, Beni Suef, Egypt

**Keywords:** Nilotinib, Ofloxacin, Capillary electrophoresis, Therapeutic drug monitoring, Real rat plasma, Biological techniques, Biotechnology, Cancer, Drug discovery, Oncology

## Abstract

Cancer is the most threatening disease worldwide. Noticeably, most cancer patients are immune-compromised, so they are more susceptible to infections. The combination of the tyrosine kinase inhibitor nilotinib (NIL) with broad-spectrum antibiotics such as the fluoroquinolone ofloxacin (OFL) is clinically rationalized based on NIL’s immunosuppressive effects and the need for infection prophylaxis in cancer patients. However, there is no published method for their in-vivo simultaneous determination in clinical biological specimens to support their pharmacokinetics and therapeutic monitoring. This study focuses on the development of a miniaturized capillary electrophoresis system-assisted with photodiode array detection (CE-PDA) for the simultaneous determination of NIL and OFL in clinical settings for analysis of plasma samples containing NIL and OFL. The conditions of electrophoretic separation of NIL, OFL, and sarafloxacin (SAR, as an internal standard), and the analytical procedures were successfully established. The electrophoretic separation of NIL, OFL, and SAR, as the internal standard, was achieved utilizing a fused silica capillary (58 cm length × 75 μm internal diameter) maintained at 25 °C using a background electrolyte solution composed of borate buffer (20 mM, pH 9.3) at a separation voltage of 30 kV. The linear range of the CE-PDA method in spiked rat plasma was determined to be 0.5–80 µg mL^− 1^ for NIL and OFL. The CE-PDA approach exhibited maximum sensitivity, with lower limits of quantitation (LLOQ) of 0.3 µg mL^− 1^ for NIL and 0.4 µg mL^− 1^ for OFL. These LLOQs were experimentally validated according to US-FDA guidlines and were lower than the calibration range’s starting point to ensure reliable quantification. In addition, the CE-PDA system was validated and subsequently applied to real plasma samples withdrawn from rats received concurrent administration of NIL and OFL. The plasma protein was precipitated by acetonitrile. The validation parameters were determined and found to comply with the requirements for the Food and Drug Administration (FDA) guidelines for validation of bioanalytical procedures. The sustainable features of the system and its environmental impact were confirmed using seven different metric tools. Finally, the proposed CE-PDA is time-saving and cost-effective, enabling its beneficial applications in the therapeutic drug monitoring of NIL and OFL in oncology clinics.

## Introduction

Cancer remains one of the leading causes of morbidity and mortality worldwide, with profound social, health, and economic implications. The global burden of cancer continues to rise due to aging populations and increasing exposure to risk factors, emphasizing the critical need for effective chemotherapeutic agents. In this context, the development and use of targeted anticancer therapies have become indispensable in prolonging survival and improving the quality of life for cancer patients.

**Fig. 1:**
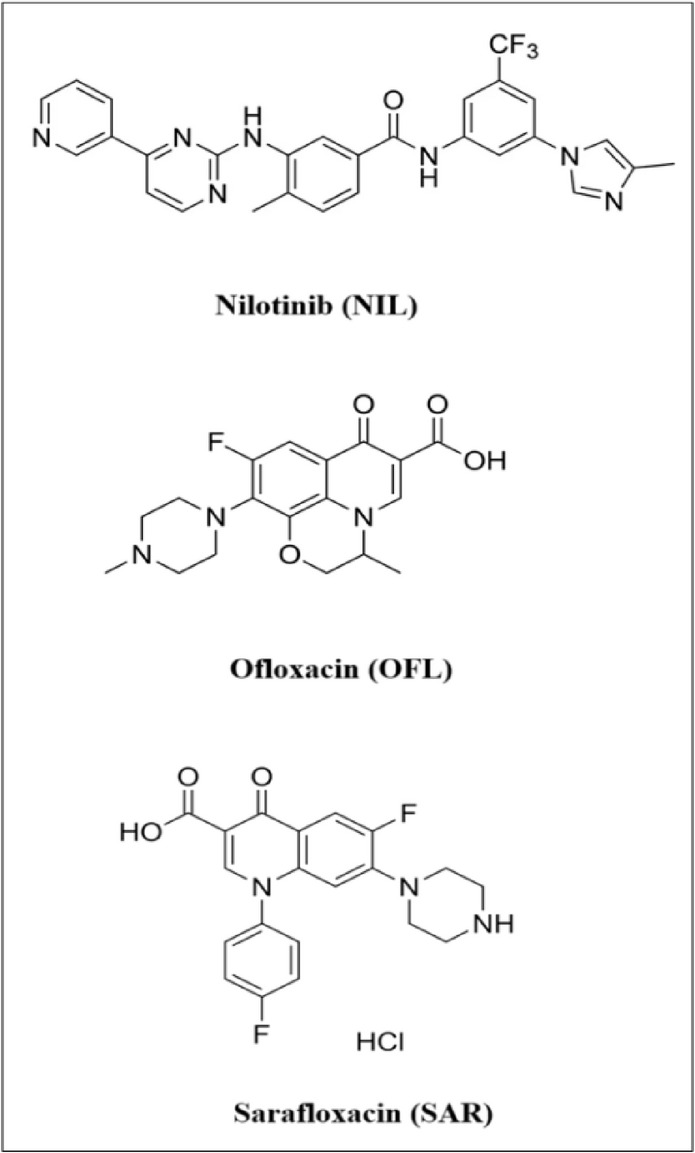
The chemical structures of nilotinib (NIL), ofloxacin (OFL) and sarafloxacin (SAR).

Nilotinib (NIL, Fig. 1) is a potent oral tyrosine kinase inhibitor that selectively targets the BCR-ABL fusion protein, a major molecular driver of chronic myeloid leukemia (CML), and is routinely employed as a first-line therapy in patients diagnosed during the chronic phase^1,2^. Despite its therapeutic advantages, NIL is known to exert immunosuppressive effects either directly or indirectly by reducing white blood cell counts, thereby increasing patient vulnerability to opportunistic infections during treatment^3^.

To mitigate these risks, broad-spectrum antibiotics such as ofloxacin (OFL, Fig. 1), a fluoroquinolone, are commonly co-administered. OFL is particularly valuable in managing or preventing infections in immunocompromised patients due to its potent antibacterial activity, favorable pharmacokinetic profile, and oral bioavailability^4–8^.

The co-administration of NIL and OFL represents a therapeutic strategy to maintain continuous cancer therapy while minimizing infection-related complications. This combination helps ensure patient stability, improves overall treatment success, and is often a necessary component of supportive care during cancer management^3–6^.To date, no published analytical method exists for their simultaneous determination in biological matrices, highlighting a significant gap addressed by this study.

Given the increasing frequency of NIL and OFL co-administration in clinical oncology, there is a growing demand for sensitive, selective, and high-throughput analytical methods capable of simultaneously quantifying both drugs in biological matrices. In this study, a capillary zone electrophoresis method was developed and validated for the simultaneous determination of NIL and OFL in rat plasma. The method presents numerous benefits, such asexcellent separation efficiency, reduced consumption of both samples and reagents, simultaneous quantification of both drugs, which is a prerequisite for conducting pharmacokinetic and drug-drug interaction studies. This approach supports both preclinical evaluations and the therapeutic monitoring of this clinically relevant drug combination. Capillary electrophoresis (CE) is particularly advantageous for such applications due to its inherent miniaturization, low solvent consumption, and compatibility with green analytical chemistry principles. Its flexibility and efficiency make it an ideal platform for developing sustainable bioanalytical methods.

NIL has been determined by several chromatographic methods either individually or in combination with other drugs. These methods include HPLC-FLU^9^, RP-HPLC^10,11^, UPLC-MS/MS^1,12^, spectrofluorimetry^13^, colorimetry^14^, voltammetry^15^, and capillary electrophoresis^16^.

OFL was also determined by numerous analytical techniques, including HPLC-UV^17,18^, LC-MS^19^, spectrophotometry^20^, spectrofluorimetry^21^, colorimetry^22^, TLC^23^, voltammetry^24^, and capillary electrophoresis^25^. All these techniques were developed for the individual determination of NIL or OFL; however, there is no published method for their in-vivo simultaneous determination. Therefore, the present study was directed towards the development of a new method for the simultaneous determination of NIL and OFL.

Capillary electrophoresis (CE) has emerged as a powerful tool in pharmaceutical and biomedical analysis due to its well-documented advantages, including minimal consumption of organic solvents and reagents, low environmental impact, cost-effectiveness, and rapid analytical throughput^26^. In particular, CE is distinguished by its short analysis time, high separation efficiency, and flexibility in method optimization, including the ability to perform parallel experiments with minimal resource demand^27^.These characteristics make CE an ideal platform for sustainable pharmaceutical analysis and therapeutic drug monitoring, especially when compared to conventional chromatographic techniques that typically consume larger volumes of organic solvents and generate more waste^28–34^. The growing emphasis on Green Analytical Chemistry has further accelerated the adoption of CE for bioanalytical applications in clinical and preclinical settings. Building on these merits, the present study is the first to report a capillary electrophoresis method coupled with photodiode array detection (CE-PDA) for the simultaneous determination of NIL and OFL in real rat plasma samples. Method development and validation were conducted in accordance with the U.S. Food and Drug Administration (FDA) guidelines for bioanalytical method validation^35^.

The CE-PDA protocol employed a simple and rapid one-step protein precipitation technique for plasma sample preparation, requiring a small sample volume and minimal use of organic solvents. The optimized method achieved complete separation of NIL and OFL in less than 12 min, demonstrating excellent resolution, precision, and accuracy. The streamlined sample pretreatment, combined with the short run time, makes this method highly suitable for high-throughput applications in clinical and preclinical laboratories. Additionally, the method exhibits strong compliance with the principles of Green Analytical Chemistry (GAC), underscoring its eco-friendly and sustainable profile. Overall, the developed CE-PDA method provides a robust, efficient and green analytical platform for the pharmacokinetic analysis, bioavailability assessment, and therapeutic monitoring of NIL and OFL in combination therapy, particularly relevant in oncology settings where co-administration is clinically warranted. In addition, the analytical approach meets the stringent requirements of both in-vitro and in-vivo bioequivalence studies to support regulatory compliance and ensure patient safety.

## Experimental

### Instrumentation

An Agilent capillary electrophoresis (CE) system (Agilent Technologies, Santa Clara, CA, USA) equipped with a photodiode array (PDA) detector was employed for all analyses. Instrument control and data acquisition were carried out using Agilent ChemStation software. Electrophoretic separations were performed using a commercially pre-deactivated fused silica capillary (total length 66 cm, effective length 58 cm, internal diameter 75 μm) obtained from Agilent Technologies (Santa Clara, CA, USA). The capillary has a factory-treated inner surface designed to reduce silanol activity and minimize analyte adsorption. Both the capillary and sample compartments were thermo stated at 25 °C, and detection was conducted at 220 nm.

The background electrolyte (BGE) consisted of borate buffer (20 mM, pH 9.3). Sample introduction was achieved by hydrodynamic injection at the anodic end under a pressure of 40 mbar for 30 s. Electrophoresis was performed at a constant voltage of 30 kV, with the cathode positioned at the detector end. To maintain reproducibility and capillary performance, a rigorous conditioning protocol was followed: prior to each injection, the capillary was sequentially flushed with 0.1 M NaOH (5 min), ultra pure deionized water (5 min), and running buffer (10 min). Between successive runs, the capillary was rinsed with deionized water (2 min) and equilibrated with BGE (4 min).

All solutions were filtered through 0.2 μm membrane filters (Millipore, Nihon, Japan) before use. A vortex mixer (Model IVM-300p, Gemmy Industrial Corp., Taipei, Taiwan) was used for sample mixing. pH adjustments were carried out using a pH meter (Model NV P-910, Consort, Belgium). Ultrapure deionized water used throughout the study was obtained from a Purelab Flex purification system (ELGA Veolia Ltd., High Wycombe, UK).

### Materials and Reagents

Reference standard materials of NIL, OFL, and the internal standard, sarafloxacin (SAR) were acquired from LC Laboratories (Woburn, MA, USA) with a purity of ≥99% as per the specified procedure. Analytical grade acetonitrile (Sigma-Aldrich, St. Louis, MO, USA) was employed for protein precipitation. Analytical grade sodium acetate trihydrate, acetic acid, and boric acid were procured from Sigma-Aldrich Chemicals Co. (St. Louis, Missouri, USA). Analytical grade sodium tetraborate decahydrate, potassium phosphate monobasic, and sodium hydroxide were acquired from Merck (Darmstadt, Germany). A borate buffer (20 mM, pH 9.3) was formulated by dissolving 0.76 g of sodium tetraborate decahydrate in deionized water and increasing the final volume to 100 mL with deionized water. The analysis utilized ultra pure deionized water and 0.2 μm membrane filters sourced from Nihon, Millipore (Yonnezawa, Yamagata, Japan). Blank rat plasma was obtained from healthy untreated rats obtained from the animal house facility of the Faculty of Pharmacy, Beni-Suef University, Egypt. All other materials utilized in this study were of analytical grade.

### Preparation of standard solutions

Standard stock solutions of NIL, OFL, and SAR were prepared by dissolving 10 mg of each drug in 10 mL of methanol, achieving a concentration of 1 mg mL^−1^. Subsequently, the stock solutions were diluted using the background electrolyte (BGE) to prepare working standard solutions for each drug at a concentration of 0.5 mg mL^−1^. The methanolic stock solutions demonstrated stability for at least three weeks under refrigeration. Internal standard selection is discussed in Sect. 3.2.

### Construction of calibration curves and method validation

Two series of test tubes were constructed with varying concentrations of NIL and OFL ranging from 0.5 to 80 µg mL^−1^in spiked rat plasma. Each sample solution was thereafter combined with 100 µL of the SAR (IS) standard working solution (0.5 mg mL^−1^), and the total volume was adjusted to 1 mL using the BGE. The solutions were administered in triplicate into the CE instrument under optimum circumstances. Calibration curves correlating detector responses (peak area ratios of analyte to internal standard) with corresponding analyte concentrations were established using these samples. Quality control (QC) samples were prepared at low, medium, and high concentration levels (20, 40, and 60 µg mL^−1^ for both NIL and OFL) across the calibration range to bracket expected plasma concentrations and comply with FDA validation guidelines.

Calibration standards for in-vivo experiments were prepared in two sets of test tubes by precisely transferring varying quantities of NIL and OFL ranging from 0.5 to 80 µg mL^−1^ from their working standard solutions (0.5 mg mL^−1^). Each sample was combined with 200 µL of blank plasma and 100 µL of the working internal standard solution (0.5 mg mL^−1^), followed by vortexing the mixtures for 1 min. To precipitate plasma proteins, the volume was adjusted to 1 mL with acetonitrile, vortexed for 1 min, subsequently subjected to centrifugation at 5,000 rpm (~ 2,830 × g) for 10 min to separate the plasma protein.

Two calibration curves were produced for each drug: one for pure standards and another for spiked plasma samples. Comparison showed minimal matrix effects, with comparable slopes and intercepts between the two curves. The spiked plasma calibration was used for quantification in in vivostudies to account for any potential matrix influence and ensure accurate measurement in biological samples. Numerous samples were generated and examined using an electrophoresis device under optimal working conditions. The peak regions of the analytes and the internal standard were documented and graphed against the respective analyte concentrations. Linear regression equations for NIL and OFL were established, enabling the determination of their concentrations in the samples. The data were subsequently employed to validate the method in accordance with FDA criteria for bioanalytical method validation^35^.

The identical process was employed to prepare quality control samples at low, medium, and high concentration levels (20, 40, 60 µg mL^−1^ for both NIL and OFL). All samples and quality control solutions were preserved at −20 °C in the freezer until the time of analysis.

### In-vivo studies in rats

Twelve Wistar albino rats, each weighing 250 ± 30 g, were obtained from the animal house facility of the Faculty of Pharmacy, Beni-Suef University, Egypt and were allocated into four groups, comprising three rats per group. All rats were maintained at a temperature of 25 °C and a relative humidity of 50%. The animals underwent the following treatments via oral gavage: Group I administered 20 mg/kg of NIL, which was dissolved in 0.5% carboxy methyl cellulose (v/v)^36^. Group II received 20 mg/kg of OFL, dissolved in 0.5% hydroxyl propyl methyl cellulose (v/v)^37^, Group Ⅲ administered 20 mg/kg of NIL and OFL which was dissolved in a mixture 0.5% carboxy methyl cellulose and 0.5% hydroxyl propyl cellulose (v/v), and Group Ⅳ was the control group.

Blood samples of approximately 400 µL were collected from each rat via the tail vein at designated times for each drug (T_max_) into heparinized tubes. Blood samples were collected from conscious rats via the tail vein at the designated time points (T_max_) using minimally invasive procedures. No anesthesia was required during blood collection, and no euthanasia (sacrifice) was performed as part of this study. All animals were handled in accordance with institutional ethical guidelines to minimize pain and distress^38,39^. Plasma samples were collected and preserved at −20 °C prior to analysis. During the analysis, plasma was thawed to room temperature. 200 µL of plasma were extracted from each sample into a clean centrifuge tube. 100µL of the internal standard’s working solution (0.5 mg mL^−1^) were added for spiking, followed by the addition of acetonitrile, a plasma protein precipitant, and the total volume was adjusted to 1 mL(resulting in a 5-fold dilution of the original plasma concentration; measured concentrations were corrected accordingly). Following a 1-minute vortex mixing step, the mixture underwent centrifugation at 5,000 rpm (~ 2,830 × g) for 20 min to isolate the plasma protein. Supernatants were aspirated and subsequently filtered using 0.45 μm syringe filters. The plasma samples were analyzed with the proposed CE-PDA system, allowing for the determination of plasma concentrations of NIL and OFL. The experiments were conducted in accordance with ethical approval protocol No. REC-H-PhBSU-022–234, issued by the Committee of Beni-Suef University, Faculty of Pharmacy, Egypt.This study is reported in accordance with the ARRIVE guidelines for reporting animal research^40^.

## Results and discussion

Capillary electrophoresis is deemed to be one of the most fundamental and widespread techniques used for separation and analysis of drugs in biological fluids owing to its high versatility^41^. This study presents a validated eco friendly electrophoretic approach for determining the co-administered drugs NIL and OFL in spiked rat plasma followed by a determination of each drug in real rat plasma at C_max_ of each drug.

### Optimization of the electrophoretic conditions

The investigation focused on examining the factors that affect the electrophoretic separation of the analytes. The objective was to obtain clearly defined and symmetrical peaks for NIL and OFL, ensuring a high signal-to-noise ratio.

#### Effect of buffer type, pH, and concentration

**Fig. 2:**
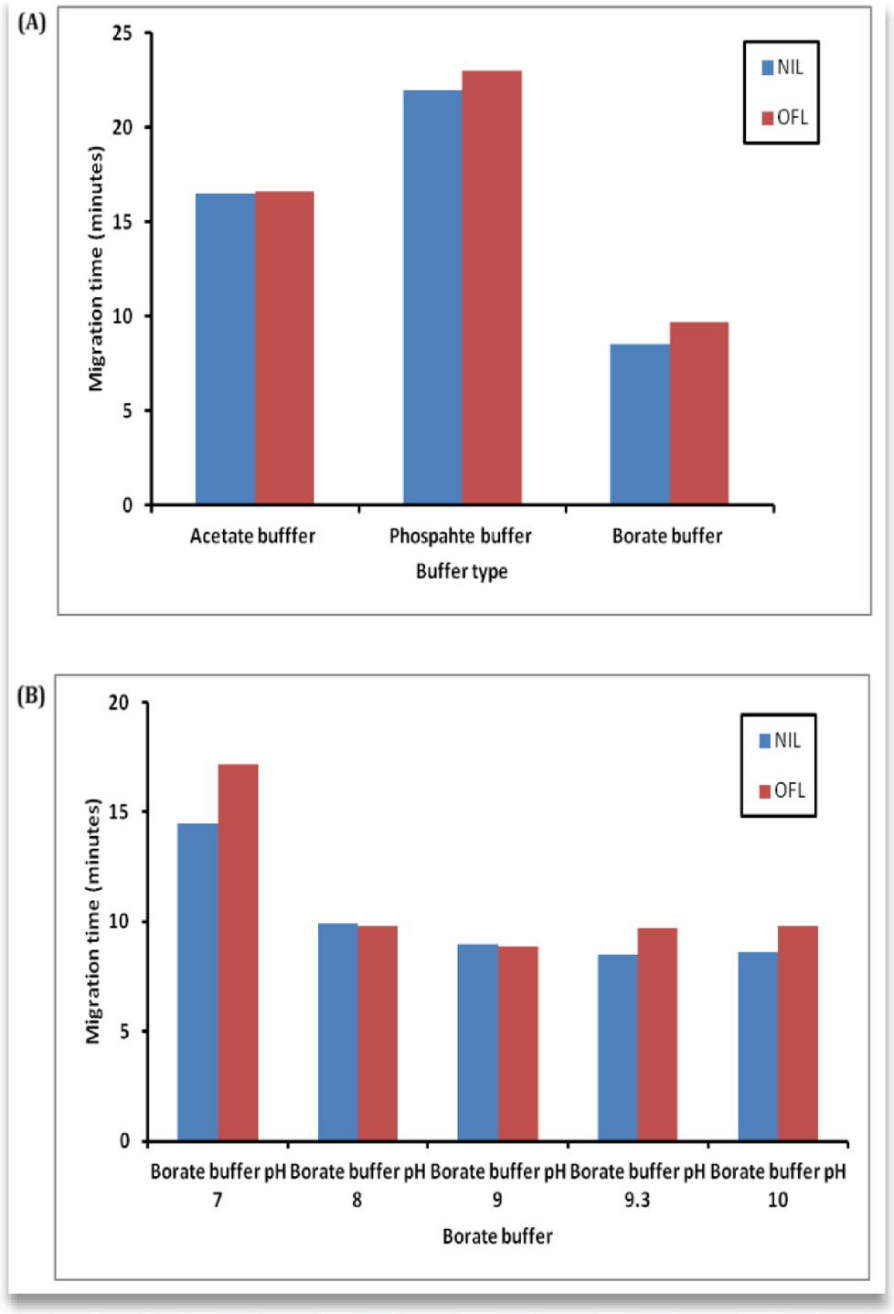
(**A**)The effect of buffer types at different pH values on the compounds’ migration time of nilotinib and ofloxacin. (**B**) The effect of borate buffer at different pH values on the compounds’ migration time ofnilotinib and ofloxacin.

In order to obtain the optimum conditions, three buffer systems namely acetate buffer (pH range 3–5), phosphate buffer (pH range 5.5–7.5), and borate buffer (pH range 7–10) were investigated. The optimum results were obtained with borate buffer in terms of peak shape, resolution, selectivity, and efficiency, as shown in Fig. 2.

The magnitude of the electroosmotic flow (EOF) is commonly influenced by the pH of the buffer solution, which influences the degree of the analyte ionization. This phenomenon could be attributed to the increased dissociation of silanol groups at the internal surface of the capillary, which results in an increase in EOF as the pH increases^42^. To examine the impact of pH on the separation of the compounds under investigation in the current study, three buffer systems were assessed as background electrolytes (BGEs) in the pH ranges of (3–10). BGE, which was initially tested, was composed of acetate buffer in the pH range of 3–5, both NIL and OFL exhibited overlapped peaks; however, BGE which was composed of phosphate buffer with pH values of 5.5–7.5 resulted in an increase in the migration time of both NIL and OFL, which eluted after 20 min. Next, borate buffers with pH values ranging from 7 to 10 were evaluated. It was observed that the migration of NIL was significantly enhanced at pH 7, but OFL was eluted after 15 min. A complete overlap between the NIL and OFL peaks was observed at pH values of 8 and 9. A complete separation between NIL and OFL peaks was achieved by increasing the pH to 9.3–10. The analysis was determined to be most effective with a borate buffer solution of pH 9.3 and consequently, this solution was selected as it generated the most symmetric and well-resolved peaks within a reasonable runtime, as shown in**(**Fig. 2B**)**.

**Fig. 3:**
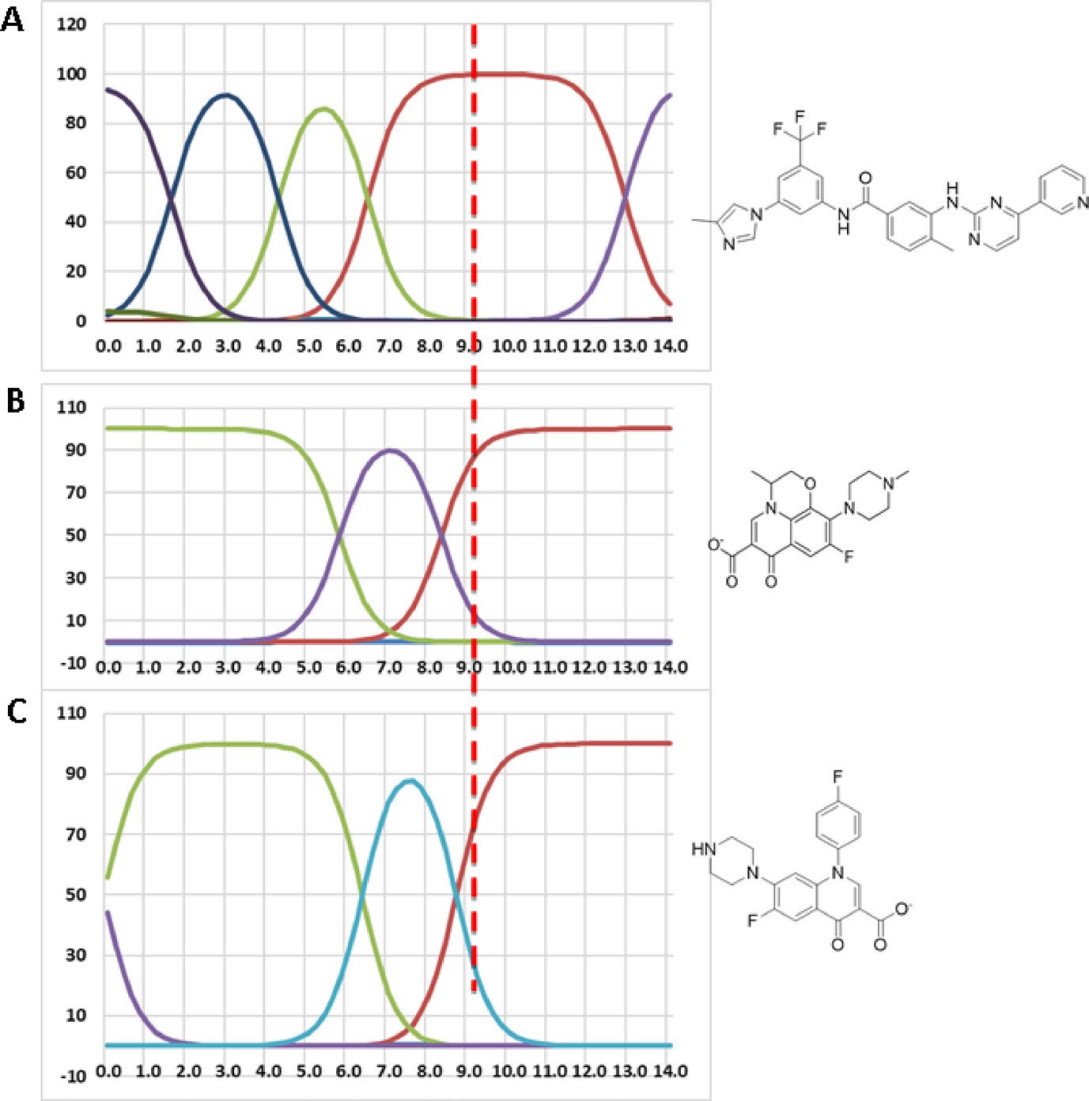
Ionization profiles of NIL, OF, and SAR at different pH values utilizing MarvinSketch. The structures are given on the right-hand side of each corresponding profile. The selection of pH 9.3 for the buffer system (vertical dashed red line) provides optimal ionization states across all compounds, enhancing the consistency and resolution of electrophoretic analysis.

A background buffer of pH 9.3 is optimally suited for the CE separation of NIL, OLF, and SAR, as it directly influences their ionization states and migration order. At this pH, NIL exists mainly in a neutral form, whereas OFL and SAR are predominantly anionic, ensuring distinct differences in electrophoretic mobilities that facilitate clear separation. Under these conditions, NIL migrates first, followed by OFL and then SAR. In CE, solute migration is determined by the combined effects of EOF, which drives all species toward the cathode, and the intrinsic electrophoretic mobility of the solutes, which depends on their charge and molecular properties. Neutral analytes, such as NIL at pH 9.3, are transported solely by EOF and thus display rapid migration. In contrast, negatively charged molecules, such as OFL and SAR, migrate against the EOF due to their electrophoretic mobility, resulting in slower net migration compared to neutral species.​ At pH 9.3, the microspecies distribution diagrams for these compounds highlights NIL’s predominance in a neutral state, while OFL and SAR possess deprotonated carboxyl groups and largely unprotonated amino groups, conferring significant negative charge (Fig. 3). This charge disparity yields considerable differences in effective electrophoretic mobilities and confirms the buffer’s efficacy for their separation. The buffer not only maintains a robust and consistent EOF, essential for efficient resolution of neutral and anionic species, but also preserves the stability of analyte ionization states, thereby enhancing overall separation reliability.

The concentration of borate buffer significantly affected the separation performance by influencing the electroosmotic flow and the current produced in the capillary. The impact of borate concentration in the running buffer was examined by altering the concentration between 5 and 50 mM. An elevation in borate concentration more than 20 mM led to diminished resolution and prolonged migration periods whereas 5 and 10 mM resulted in overlaid peaks. To reduce analysis time while preserving optimal resolution, a borate buffer concentration of 20 mM was chosen.

#### Effect of applied voltage

The effectiveness of capillary electrophoretic separations is greatly affected by the applied voltage^43^.Typically, enhancing the applied voltage can lead to improved resolution. A sequence of experiments was carried out, incrementally increasing the applied voltage from 15 to 30 kV. With the rise in voltage, there was a corresponding decrease in migration time, leading to an enhancement in peak shape. Nevertheless, lowering the applied voltage beneath 30 kV led to a decrease in separation efficiency. The application of a voltage of 30 kV resulted in effective separations, achieving a migration time of less than 12 min. Consequently, a voltage of 30 kV was chosen as it offered the most advantageous balance between peak resolution, peak response, and analysis duration of less than 12 min for the analytes.

#### Effect of injection time and pressure

A range of durations, particularly from 30 to 90 s, was examined to identify the optimal sample injection period. The injection duration was found to have a minimal influence on the migration time of the medications; however, it played a significant role in the resolution of the analyzed components^44^. Minimal difference in resolution was observed when injection durations ranged from 40 to 60 s. Extending the injection time beyond that range led to increased sensitivity, as evidenced by lower limits of detection and quantitation; however, this came at the cost of a noticeable reduction in resolution. As a result, a 30-second injection period was selected for the analysis.

The pressure methodically varied between 20 and 100 mbar to assess the impact on the separation process. Enhancing the pressure led to improved efficiency and a reduction in migration time. A pressure of 40 mbar and an injection period of 30 s were chosen for further analysis. The selected parameters aim to achieve a balance between optimal separation, well-defined peak shapes, and maintaining an analysis time of less than 12 min for the analytes.

#### Effect of capillary temperature

Maintaining precise control of capillary temperature is essential for achieving consistent results in the analysis. A segment of the electrical energy flowing through the capillary underwent conversion into Joule heating. The influence of temperature on buffer viscosity is crucial, as it directly impacts the migration durations and velocities of the analytes, thereby affecting resolution^45^. Effective temperature management is crucial to reduce the effects of Joule heating. As the temperature rose from 15 to 50 °C, a decrease in migration duration was noted, resulting in overlapping peaks at temperatures exceeding 25 °C. This phenomenon can be linked to a reduction in the viscosity of the background electrolyte as the temperature rises. The analysis revealed that an optimal temperature of 25 °C was established based on these findings.

#### Effect of detecting wavelength

Electropherograms were compared at different wavelengths including the characteristic absorption maxima for each compound (210, 220, 254, 300 nm) to select the wavelength offering the highest sensitivity for simultaneous detection of all analytes with minimal baseline noise. A detection wavelength of 220 nm was chosen as it yielded the highest response for all analytes.

Table 1 summarizes the optimization of different conditions affecting the CE method, and the established optimum values.

**Table 1:** Optimization of experimental conditions for the CE-PDA for the simultaneous determination of NIL and OFL.

Parameter investigated	Studied range	Optimum value
Buffer pH (unit)	3 to 10	9.3
Buffer concentration (mM)	5–50 mM	20 mM
Applied voltage (kV)	15–30 kV	30 kV
Injection time (seconds)	30–90 s	30 s
Pressure (mbar)	20–100 mbar	40 mbar
Temperature (°C)	15–50 C°	25 C°

### Internal standard selection and system suitability

Sarafloxacin (SAR, Fig. 1) was selected as the internal standard due to its structural similarity to fluoroquinolone analytes, comparable pKa values, and similar amphoteric behavior under alkaline electrophoretic conditions. SAR demonstrated stable extraction recovery using the same protein-precipitation procedure applied to NIL and OFL and produced sharp, well-defined peaks without co-migration with either analyte. Its migration time consistently appeared after NIL and OFL, allowing reliable correction of injection variability and instrumental fluctuations, making it an optimal internal standard for the proposed CE–PDA method.

Under the optimized conditions, the system suitability parameters were calculated, summarized and presented in Table 2 to test the overall performance of the method.

**Table 2:** System suitability parameters of NIL and OFL by CE-PDA method.

Analyte	Migration time (min.)	Number of theoretical Plates	Relative Migration(*R*_m_)^a^ (1–10)	Selectivity(α)^b^ (> 1)	Resolution (*R*_s_)^c^ (> 1.5)	Tailing factor (T_f_)^d^ (0.9–1.2)
**NIL**	8.5 ± 0.014	51,378	1.66			0.92
				1.20	7.33	
**OFL**	9.6 ± 0.070	65,536	2.00			1.05
				1.17	7.33	
**SAR**	10.7 ± 0.074	81,415	2.34			1.12

^**a**^**Relative Migration (R**_**m**_**)**:R_m_ = (t_R_ - t_0_)/t_0_, where t_R_ is the migration time of the analyte and t0 is the migration time of the neutral marker.

^**b**^**Selectivity (α): α** = R_m2_/R_m1_.

^**c**^**Resolution (Rs)**:Rs = 2(t_R2_ - t_R1_)/(w_1_ + w_2_).

^**d**^**Tailing factor (T**_**f**_**)**:T_f_ = w_0.05_/2f, where w_0.05_is the peak width at 5% height and f is the distance from the peak front to the peak maximum at 5% height.

All calculations were performed using ChemStation software (Agilent Technologies).

To summarize, the optimal separation of analytes was successfully attained utilizing a fused silica capillary featuring an effective length of 58 cm and an inner diameter of 75 μm. The background electrolyte was composed of a borate buffer at a concentration of 20 mM and a pH of 9.3, maintained at a temperature of 25 °C. The conditions utilized for the separation comprised a voltage of 30 kV, a pressure of 40 mbar, an injection time of 30 s, and a wavelength of 220 nm. The data is presented in Table 1.

**Fig. 4:**
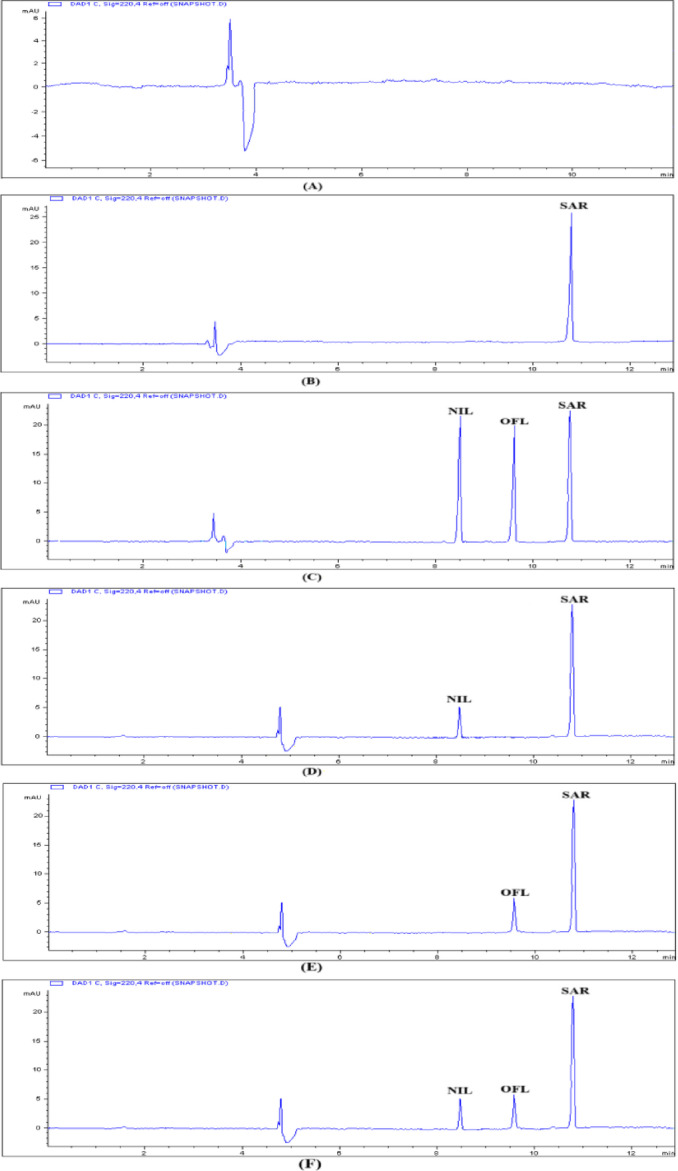
Capillary electropherograms of (**A**) blank plasma, (**B**) plasma sample spiked with 50 µg mL^− 1^ of IS, (**C**) plasma sample spiked with 50 µg mL^− 1^ IS, 40 µg mL^−1^nilotinib, 30 µg mL^−1^ofloxacin, (**D**) real rat plasma sample after administration of nilotinib, spiking with 50 µg mL^−1^IS,**(E)** realrat plasma sampleafter administration of ofloxacin after spiking with 50 µg mL^−1^IS and **(F)** realrat plasma sample after administration of nilotinib and ofloxacin after spiking with 50 µg mL^−1^IS.

Figure 4 displays a representative electropherogram of a standard mixture of NIL and OFL obtained through the optimized method. The observed peaks demonstrated distinct sharpness and full baseline separation. The approach facilitated sufficient resolution of the components within an appropriate analysis timeframe. The elution times recorded for NIL and OFL were 8.5 ± 0.014 and 9.6 ± 0.070 min, respectively.

The system suitability parameters are summarized in Table 2. The CE-PDA system exhibited acceptable resolution, demonstrating sufficient migration factors and remarkable efficiency, as evidenced by a significant number of theoretical plates. The assessment of peak asymmetry was calculated using the tailing factor, which remained below the critical value of 1.2^46^, thereby confirming an acceptable level of peak asymmetry.

### Method validation

The CE-PDA method was validated in accordance with US-FDA guidelines for bioanalytical method validation, focusing on the parameters which specified below.

#### Linear range

In order to evaluate the linearity of the CE-PDA method, a series of 10 concentration levels of samples were analyzed to produce the calibration plots. A linear regression analysis using ordinary least squares (OLS) regression was performed depending on the data set, resulting in the computation of linear fitting equations and their corresponding correlation coefficients. The linearity of CE-PDA for NIL and OFL was assessed through correlation coefficients within the designated concentration ranges. The linear range (0.5–80 µg mL⁻¹) and the sensitivity limits (LOD and LLOQ) were established relative to the original plasma concentration. This approach ensures that all reported limits fully incorporate the 5-fold dilution factor utilized during the protein precipitation and sample preparation protocol. Table 3 presents a summary of the calibration parameters for both NIL and OFL. The method demonstrated linearity, exhibiting excellent correlation coefficients, within concentration ranges of 0.5 to 80 µg mL⁻¹ for both NIL and OFL (Table 3).

**Table 3:** Validation parameters of CE-PDA for the simultaneous determination of NIL and OFL.

Parameters	NIL	OFL
Calibration range (µg mL^− 1^)	0.5–80	0.5–80
Slope	1.8911	1.9609
Intercept	−1.3915	−2.784
Correlation coefficient (r)	0.9998	0.9996
LOD (µg mL^− 1^)	0.12	0.13
LLOQ (µg mL^− 1^)	0.3	0.4

#### Sensitivity

The sensitivity of CE-PDA was evaluated and reported in terms of its limits of detection (LOD) and lower limits of quantification (LLOQ). The LOD was determined based on a signal-to-noise ratio of 3:1, whereas the LLOQ was experimentally established as the lowest concentration achieving acceptable accuracy (80–120%) and precision (RSD ≤ 20%) in accordance with US-FDA bioanalytical method validation guidelines. The calibration range starts at 0.5 µg mL⁻¹, which is above the LLOQ, to ensure robust quantification in routine analysis. The LOD values for NIL and OFL were 0.12 and 0.13 µg mL⁻¹, respectively, while the LLOQ values were 0.3 and 0.4 µg mL⁻¹ for NIL and OFL, respectively **(**Table 3**)**. These LLOQ values demonstrate sufficient sensitivity of the CE-PDA method for quantifying NIL and OFL in plasma samples of treated subjects, with reported Cmax values of 0.63 and 0.88 µg mL⁻¹ for NIL and OFL, respectively, (Table 3)^36,37^.

#### Selectivity

The specificity assessment involved a comparison of electropherograms derived from three groups of plasma samples. The groups comprised blank plasma, plasma spiked with NIL, OFL, and IS, as well as plasma samples from rats administered combined doses of NIL and OFL, subsequently spiked with IS. The electropherograms (Fig. 4) indicate the absence of peaks from the plasma matrix at the migration times of NIL, OFL, or IS, thereby confirming the selectivity of the CE-PDA method for the simultaneous detection of NIL and OFL in theplasma samples.

#### Accuracy and precision

An evaluation of accuracy and precision was performed for intra-day and inter-day measurements. Five replicates were examined for both NIL and OFL across three concentration levels: low quality control (LQC), medium quality control (MQC), and highquality control (HQC). The intra-day accuracy, indicated as bias percent, varied between − 4.69% and 3.11%, whereas the inter-day accuracy fluctuated from − 3.21% to 2.87% (Table 4). Precision was assessed through relative standard deviation (RSD) percentages, revealing intra-day precision between 1.662% and 4.250%, and inter-day precision between 1.257% and 5.344%. The minimal bias and RSD values validate the accuracy and precision of CE-PDA, respectively. All values were within FDA acceptance criteria.

**Table 4:** The intra– and inter–day precision and accuracy of the proposed CE-PDA for the simultaneous determination of NIL and OFLin spiked rat plasma samples.

Concentration (µg mL^− 1^)	Intra–day			Inter–day		
	Recovery (%) ^a^	RSD (%) ^a^	Bias (%) ^b^	Recovery(%) ^a^	RSD (%) ^a^	Bias (%) ^b^
NIL						
20	99.50	2.176	−0.50	98.24	2.417	−1.76
40	100.83	1.695	0.83	97.55	3.231	−2.45
60	97.12	2.539	−2.88	100.20	1.257	0.20
OFL						
20	99.43	3.641	−0.57	99.86	2.162	−0.14
40	96.17	2.251	−3.83	98.04	3.326	−1.96
60	95.31	1.662	−4.69	96.79	3.462	−3.21

^a^ Average of 5 determinations.

^b^ Bias = [(measured concentration - nominal concentration)/nominal concentration] ⋅ 100. Values are an average of 3 determinations.

#### Stability and extraction recovery

The stability of samples with three concentration levels of NIL and OFL was evaluated through multiple stability methods, including storage at room temperature (25 °C) for 6 h (bench-top stability) and exposure to three freeze-thaw cycles from − 20 °C for 12 h to room temperature (freeze and thaw stability). The recoveries(*n* = 5) achieved were varied from 96.48 ± 3.29% to 105.47 ± 3.14% for NIL and from 96.28 ± 2.11% to 102.39 ± 2.43% for OFL (Table 5). The results demonstrate that the plasma matrix does not influence the stability of NIL and OFL in the prepared samples under the specified analysis conditions.

**Table 5:** Stability results of NIL and OFL in spiked rat plasma at different conditions using the proposed CE-PDA.

Concentration (µg mL^− 1^)	Recovery (%± RSD)^a^	
	Three freeze thaw cycles ^b^	Bench top stability
NIL		
20	105.47	103.17
40	98.46	98.26
60	100.28	96.48
Mean ± % RSD	101.40 ± 3.587	99.30 ± 3.489
OFL		
20	102.39	96.28
40	99.25	98.04
60	97.21	101.29
Mean ± % RSD	99.62 ± 2.619	98.54 ± 2.579

^a^Average of 5 determinations.

^b^ Freezing was done at − 20 °C.

The extraction efficiency was evaluated, yielding mean extraction recoveries of 101.36 ± 1.836% (SD)for NIL and 99.99 ± 2.012%(SD) for OFL (Table 6). The results validate the efficacy of the extraction procedures utilized.

**Table 6:** The extraction recovery results of NIL and OFL in spiked rat plasma.

Concentration (µg mL^− 1^)	Recovery (%) ^a^	
	NIL	OFL
20	101.28	102.18
40	99.54	98.22
60	103.26	99.58
Mean ± % SD	101.36 ± 1.836	99.99 ± 2.012

^a^ Average of 3 determinations.

### Utilization of CE-PDA for the analysis of real plasma samples from rats

The developed CE–PDA method was applied to analyze real plasma samples collected from rats following oral administration of NIL alone, OFL alone, and the combined NIL and OFL co-administered dose. This experimental design enabled assessment of both single-drug pharmacokinetic exposure and the simultaneous quantification of NIL and OFL under co-administration conditions. Literature reports indicate C_max_ values of approximately 0.63 µg mL⁻¹ for NIL and 0.88 µg mL⁻¹ for OFL at comparable dosing levels^36,37^. These reference values were used qualitatively to verify whether the measured plasma concentrations fall within pharmacologically relevant ranges.

Representative electropherograms for each group are shown in Fig. 4, demonstrating clear resolution of NIL, OFL, and the internal standard SAR, with no evidence of endogenous interference. The method maintained excellent selectivity even in the co-administered plasma samples, confirming its robustness for simultaneous bioanalysis. The measured plasma concentrations for all treatment groups are presented in Table 7. For single-dose administration, NIL concentrations ranged from 0.58 to 0.66 µg mL⁻¹, and OFL concentrations ranged from 0.68 to 1.12 µg mL⁻¹, in good agreement with their expected C_max_ values.

**Table 7:** Results of determination of NILand OFLin real rat plasma^a^.

Group	Sample	Expected conc. (µgmL^− 1^)	Found NIL (µgmL^− 1^)	Found OFL (µgmL^− 1^)
NIL single-dose	1	0.63	0.58	—
NIL single-dose	2	0.63	0.66	—
NIL single-dose	3	0.63	0.61	—
Mean ± SD			0.62 ± 0.041	—
OFL single-dose	1	0.88	—	0.68
OFL single-dose	2	0.88	—	1.12
OFL single-dose	3	0.88	—	0.84
Mean ± SD			—	0.88 ± 0.221
NIL + OFL Co-admin	1	0.63/0.88	1.09	1.10
NIL + OFL Co-admin	2	0.63/0.88	1.15	1.16
NIL + OFL Co-admin	3	0.63/0.88	1.12	1.13
Mean ± SD			1.12 ± 0.030	1.13 ± 0.324

^a^Reported values represent concentrations in the original plasma matrix. The 5-fold sample preparation dilution was fully accounted for via back-calculation using the validated regression equations.

Importantly, rats receiving the combined NIL and OFL dose exhibited significantly higher systemic exposure to both drugs, with NIL increasing by approximately 81% (mean 1.12 µg mL⁻¹) and OFL increasing by approximately 28% (mean 1.13 µg mL⁻¹) compared with the corresponding single-dose groups. It is noteworthy that despite the 5-fold dilution utilized during sample preparation, the method remained highly sensitive for these determinations. For instance, the measuredC_max_ for NIL (~ 1.12 µg mL⁻¹) results in an “on-capillary” concentration of approximately 0.22 µg mL⁻¹ after dilution. This value is nearly three times higher than the system’s functional “on-capillary” LOQ (~ 0.07 µg mL⁻¹), providing a substantial safety margin that ensures the accuracy and reliability of the reported peak plasma concentrations. All co-administration concentrations remained within the validated calibration range, which was established relative to the original plasma matrix to fully incorporate the dilution factor. These marked increases in systemic exposure strongly suggest a possible drug–drug interaction between NIL and OFL, warranting further mechanistic in-vivo and in-vitro investigations and providing initial data for future pharmacokinetic studies and therapeutic drug monitoring.

### Comprehensive greenness and sustainability evaluation

Green Analytical Chemistry (GAC) promotes the development of environmentally responsible analytical methodologies that minimize the use and generation of hazardous substances while maintaining high analytical performance, even at trace analyte levels in complex biological matrices^47^. To comprehensively evaluate the sustainability profile of the developed CE–PDA method, seven complementary greenness and sustainability assessment tools were employed: the Analytical Eco-Scale Assessment (ESA)^48^, the Green Analytical Procedure Index (GAPI)^49^, the Analytical GREEnness metric (AGREE)^50^, the Blueness Analytical Greenness Index (BAGI)^51^, the RGB model for White Analytical Chemistry (WAC)^52^, the Analytical GREEnness metric for Sustainability Assessment (AGSA)^53^, and the Carbon Footprint Reduction Index (CaFRI)^54^.

#### Classical greenness evaluation tools

The Analytical Eco-Scale Assessment (ESA) quantifies the environmental burden of an analytical procedure by assigning penalty points for hazardous reagents, energy use, occupational risks, and waste generation. These points are subtracted from an ideal score of 100 to yield a final greenness value, where higher scores indicate better environmental performance. The proposed CE–PDA method achieved an ESA score of 87, confirming excellent greenness and minimal ecological burden. This strong performance stems from the inherently low solvent and reagent consumption of the CE platform and the use of an aqueous borate buffer as the background electrolyte(Table 8).

**Table 8:** ESA, GAPI, AGREE, BAGI, AGSA, RBG and CaFRI tools for assessment of greenness values of the proposed CE-PDA.

Eco-scale assessment								
Reagents								
Solvents		Amount		Hazard^a^			Total penalty points^b^	
**Acetonitrile**		1(< 10 mL)		2 (2 pictogram, danger)			4	
**Sodium tetraborate decahydrate**		1(0.7 ~ < 10 mL)		2 (1 pictogram, danger)			2	
**Instruments**								
**Energy used**		1(≤ 1.5 kWh per sample)						
**Occupational hazard**		0						
**Waste**^**c**^, **Waste treatment**		3(1–10 mL), 3 (no waste treatment)						
**Total penalty points**		Σ13						
**Eco-scale score**		87						

**GAPI pictogram**		**AGREE pictogram**		**BAGI pictogram**			**AGSA pictogram**	
			

**RGB**				**CaFRI pictogram**				
	

^a^ Hazard penalty points = No. of pictograms × signal. The signal maybe warning = 1 or danger = 2.

^b^The total penalty points = the amount penalty points ×hazard penalty points. ^c^Waste =flow rate × run time.

The Green Analytical Procedure Index (GAPI) provides a holistic, color-coded evaluation across the entire analytical workflow, covering five major stages: sample collection, preparation, reagent use, instrumentation, and waste management. The GAPI pictogram for the CE–PDA method exhibited five green, six yellow, and four red zones, signifying a low overall environmental impact. The few red segments were primarily linked to the use of small amounts of acetonitrile for plasma protein precipitation which is a necessary compromise to ensure efficient deproteinization (Table 8).

The Analytical GREEnness metric (AGREE) assesses compliance with the twelve principles of GAC and provides a single composite score between 0 and 1. The CE–PDA method achieved an AGREE score of 0.69, demonstrating good adherence to principles such as miniaturization, reduced waste generation, and short analytical run times (Table 8).

#### Practical and functional evaluation: blueness analytical greenness index (BAGI)

While the preceding tools focus primarily on environmental aspects, the Blueness Analytical Greenness Index (BAGI) extends the sustainability assessment to encompass practical feasibility and operational efficiency. It emphasizes that a truly sustainable analytical method must be both environmentally sound and economically and functionally viable for real-world laboratory application.

The BAGI model quantitatively evaluates ten operational and performance-related parameters, including instrumental complexity, ease and safety of operation, analysis time, cost, automation potential, versatility, robustness, waste management, energy consumption, and long-term operational stability. Each criterion is scored on a normalized scale and integrated into both a numerical BAGI score and an asteroid-shaped pictogram, where broader expansion indicates superior sustainability performance.

The CE–PDA method achieved a BAGI score of 80 (Table 8), placing it within the excellent blueness category. This high score reflects its short analysis time (< 12 min), low operational cost, and reduced reagent consumption, all of which enhance analytical throughput and efficiency. The simplicity of the CE setup and its automated capillary rinsing cycles minimize cross-contamination, sample carryover, and human error which is key contributors to method robustness. Furthermore, the CE system’s potential for automation and miniaturization aligns with modern trends in high-throughput pharmacokinetic analysis, enabling faster sample turnover compared with conventional HPLC or LC–MS systems.

Unlike chromatographic methods that depend on continuous solvent pumping and pressure-driven flow, CE operates with minimal energy demand and negligible solvent waste, offering a distinct advantage in terms of operational sustainability. These features collectively justify the high BAGI score and demonstrate that the developed CE–PDA method is not only green but also “blue” that is practically feasible, cost-effective, and highly adaptable to routine analytical environments.

#### Holistic white analytical chemistry evaluation (RGB Model)

The White Analytical Chemistry (WAC) approach, based on the RGB 12-criteria model, was applied to ensure that the developed CE–PDA method achieves a balanced compromise between analytical performance, environmental sustainability, and practical applicability. In this holistic model, each domain is equally weighted: the Red component (R) represents analytical efficiency and validation quality; the Green component (G) reflects the environmental and safety impact; and the Blue component (B) evaluates economic and practical feasibility. The CE–PDA method exhibited excellent overall harmony, with an RGB score of (105, 77.5, and 103.8) and a calculated Whiteness value of 95.4% (Table 8), confirming that the method is not only analytically powerful but also environmentally and operationally sound. The high Red score emphasizes the method’s superior precision, accuracy, and wide analytical range for simultaneous determination of NIL and OFL. The moderate Green score reflects minimal solvent and reagent consumption inherent to CE, with minor deductions due to the use of acetonitrile and in vivo validation. Meanwhile, the strong Blue score underscores the method’s practicality, speed, and low cost. Collectively, these results demonstrate that the proposed CE–PDA method represents a white, sustainable, and high-performance analytical approach, fully aligned with the principles of modern green and white analytical chemistry.

#### Advanced sustainability metrics: AGSA and CaFRI

To deepen the assessment, two advanced quantitative sustainability models were applied: the Analytical GREEnness metric for Sustainability Assessment (AGSA) and the Carbon Footprint Reduction Index (CaFRI).

The AGSA tool provides a holistic, semi-quantitative visualization of the method’s environmental sustainability across twelve green chemistry principles. The method achieved an impressive AGSA score of 91.67, reflecting its strong compliance with the eco-friendly analytical guidelines. As illustrated in the AGSA diagram (Table 8), nearly all criteria were rated in the green zone, confirming the method’s minimal ecological impact. The outstanding scores were particularly attributed to the very low solvent and reagent consumption, the use of an aqueous borate buffer as the background electrolyte, and the generation of negligible chemical waste per analysis. The method’s short run time (< 12 min) and absence of derivatization or hazardous reagents further enhanced its environmental profile. Slight deductions in the AGSA evaluation were related to the limited use of acetonitrile during plasma protein precipitation and the in vivo validation phase. Overall, the obtained AGSA score positions the proposed CE–PDA method among the most sustainable analytical techniques, aligning perfectly with the principles of Green and White Analytical Chemistry and reinforcing its suitability for pharmacokinetic and bioanalytical applications.

The environmental sustainability of the developed CE–PDA method was assessed using the Carbon Footprint Reduction Index (CaFRI), a newly introduced greenness metric that quantitatively estimates the reduction in carbon emissions associated with analytical processes. The obtained CaFRI score of 86 reflects an excellent environmental performance, positioning the method within the green category of the CaFRI scale. This high score arises from the technique’s minimal electrical energy demand, elimination of continuous solvent pumping, and very low consumption of organic reagents, as only micro-volumes of acetonitrile were used for one-step plasma protein precipitation. Moreover, the use of an aqueous borate buffer as the background electrolyte and the generation of negligible chemical waste further reduced the method’s carbon footprint. Moderate penalties were assigned to the limited use of acetonitrile and the cooling during biological validation. Nonetheless, the overall score clearly confirms that the CE–PDA platform substantially minimizes laboratory-scale greenhouse gas emissions compared with conventional chromatographic procedures. Integrating the resulted high score highlights its strong compliance with both Green Analytical Chemistry and sustainable carbon-management principles, supporting its designation as a low-impact, eco-efficient analytical technology for pharmacokinetic and therapeutic monitoring studies.

#### Overall assessment

The combined application of seven independent greenness and sustainability assessment tools: ESA, GAPI, AGREE, BAGI, RGB, AGSA, and CaFRI provides a multidimensional and robust evaluation of the developed CE–PDA method. The consistent outcomes across all metrics demonstrate that the method is highly sustainable and environmentally benign, meeting the rigorous standards of both Green Analytical Chemistry (GAC) and White Analytical Chemistry (WAC). Overall, the proposed CE–PDA method stands as a sensitive, sustainable, and operationally efficient analytical approach for the simultaneous determination of NIL and OFL in plasma, offering a low-impact alternative ideally suited for pharmacokinetic and therapeutic-drug-monitoring applications in oncology clinics.

## Comparison of the proposed CE-PDA with reported methods

The reported methods for the determination of NIL and OFL were reviewed, and the main performance parameters were summarized in Table 9. Based on the comprehensive comparison, the proposed CE-PDA method demonstrates distinct and compelling advantages for the simultaneous determination of NIL and OFL in rat plasma, particularly when benchmarked against existing reported methods. The main advantageous points are summarized in the following:

**Table 9:** Comparison of the proposed CE-PDA with reported methods.

Technique	Analytes	Matrix	Sensitivity expressed as LOQ (µgmL⁻¹)	Linearity (µgmL⁻¹)	Solvent/sample prep (summary)	Ref
The proposed method: CE–PDA	NIL and OFL	Rat plasma	For NIL:0.3* For OFL:0.4*	0.5–80 for NIL and OFL	BGE : borate buffer at pH: 9.3, minimal organic solvents (only acetonitrile for protein precipitation).	P.S**
RP-HPLC	OFL	Human plasma	0.5	0.5–6.5	Organic solvents are mainly used.	^55^
Spectrophotometry	OFL	NA	0.96	1–30	Water often used for sample preparation for tablets.	^56^
Spectrophotometry	OFL	NA	1.82	2–30	Water often used for sample preparation for tablets.	^56^
RP-HPLC	OFL	Dosage form	6	2–40	Organic solvents are mainly used.	^57^
HPTLC	OFL	Dosage form	NA	0.02–0.1	Organic solvents are mainly used.	^57^
LC-MS/MS	OFL	Human plasma	0.078*	0.078–20.078	Organic solvents are mainly used.	^58^
UPLC-MS	OFL	Aqueous humor	0.10	0.1–8.1	Organic solvents are mainly used.	^59^
RP-HPLC	NIL	Dosage form	NA	5–250	Organic solvents are mainly used.	^60^
Spectrophotometry	NIL	Dosage form	0.85	7–12	Water and methanol (1:1).	^61^
UPLC- MS/MS	NIL	Human plasma	0.0024*	0.0024–4.7.0024.7	Organic solvents are mainly used.	^1^
RP-UFLC	NIL	NA	0.1897	0.2–80	Organic solvents are mainly used.	^62^
HPLC-UV	NIL	Human plasma	0.125	0.125–7.125	Organic solvents are mainly used.	^63^
HPLC-UV	NIL	Human plasma	0.5	0.5–24	Organic solvents are mainly used.	^64^
Spectrophotometry	NIL	Human plasma	0.68	1–9	The technique depends on the reaction of amine group with erythrosine B in the Britton-Robinson buffer.	^65^
Spectrophotometry	NIL	Human plasma	0.026	0.04–0.7	The technique depends on measuring the quenching of the native fluorescence of erythrosine B by adding NIL in acidic medium.	^65^

*LLOQ.

**The proposed study.

### Uniqueness in simultaneous analysis

A key differentiator of the proposed CE method is that it is the first and only method developed for the simultaneous quantification of NIL and OFL. All other methods listed are designed for the analysis of either NIL or OFL individually, often in combination with different drugs. This simultaneous capability is highly valuable for applications where both analytes are present, enhancing analytical efficiency.

### Superior sensitivity and applicability

The method exhibits excellent sensitivity, which is crucial for therapeutic drug monitoring (TDM) in biological fluids. Obviously, the linearity of the method covers a wide range of 0.5–80 µgmL⁻¹ for both analytes. Additionally, The LOD and LLOQ values for both NIL (0.12 and 0.30 µgmL⁻¹) and OFL (0.13 and 0.40 µgmL⁻¹) are sufficiently low to detect these drugs at their maximum plasma concentration (C_max_), a requirement where several other methods^55–57,61,64,65^ fail due to inadequate sensitivity.While online preconcentration techniques could further enhance sensitivity, the achieved LOQ was adequate for this proof-of-concept study at reported C_max_ levels.

### Simplified and greener sample preparation

The sample preparation protocol for the CE method is notably simpler and more environmentally friendly compared to most chromatographic and mass spectrometric techniques. For solvent consumption: the CE method uses a borate buffer at pH 9.3 as the BGE, with minimal organic solvent (only acetonitrile for protein precipitation). In stark contrast, the majority of compared methods (HPLC, UPLC, LC-MS/MS, HPTLC) rely heavily on large volumes of organic solvents in their mobile phases, which increases cost, toxicity, and environmental impact. For matrix compatibility: The method is directly applicable to complex rat plasma samples. The CE system’s open-tubular design minimizes issues like column fouling, which is a common problem in HPLC methods for biological samples^63,64^, often necessitating extensive and solvent-intensive sample pre-treatment like solid-phase extraction.

### Exceptional greenness profile

The “green” character of the proposed CE method is a major advantage highlighted throughout the comparison. The CE method features low solventconsumption, low waste generation, and relatively low energy demands compared to techniques like LC-MS/MS^57^ and UPLC-MS^58^, which, while highly sensitive, consume significant amounts of organic solvents and energy and require expensive gases.This strong greenness profile is quantitatively supported when compared to a reported HPLC-UV method^64^, which, despite having a greenness assessment, achieved lower scores (AGREE: 0.59, BAGI: 75.0, RGB: 75.7) than the proposed CE method.

### Balanced performance and practicality

While highly sensitive techniques like UPLC-MS/MS^1^ offer lower LOQs, they do so at the cost of high instrument and operational expense, complex maintenance, and a poorer environmental footprint. The proposed CE method strikes an optimal balance, providing the necessary sensitivity for TDM in a biological matrix, with high biological relevance, simplicity, and a superior green chemistry profile.

In conclusion, the developed CE-PDA method is not only unique for its simultaneous analysis of NIL and OFL but also stands out for its appropriate sensitivity, straightforward sample preparation, and excellent environmental friendliness, making it a superior and sustainable alternative to the existing methodologies.

## Conclusion

For the first time, a sensitive, selective, and environmentally friendly capillary electrophoresis method with photodiode array detection (CE-PDA) has been developed and validated for the simultaneous determination of the anticancer drug nilotinib (NIL) and the antibiotic ofloxacin (OFL) in plasma samples. The method presented several analytical advantages, including a simple one-step protein precipitation for sample preparation, reduced sample and solvent consumption, high separation efficiency, and a short analysis time, making it well-suited for high-throughput applications. Its streamlined workflow enables rapid processing of multiple samples, supporting routine use in clinical and preclinical laboratories.Validation according to FDA bioanalytical guidelines confirmed the method’s robustness; stability results ranged from 96.28% to 105.47%. The method demonstrated high levels of accuracy and precision; the **accuracy (expressed as bias %)** ranged from **− 4.69% to 3.11%**, while **precision (RSD %)** remained between **1.257% and 5.344%**. These validated parameters support the method’s application in streamlining sample detection in pharmacokinetic and bioequivalence studies. Application in rat models enabled reliable determination of C_max_ values for both drugs, highlighting its utility in therapeutic drug monitoring. The method enables simultaneous quantification of both drugs, which is a prerequisite for conducting pharmacokinetic and drug-drug interaction studies in human subjects. Greenness evaluation confirmed strong compliance with Green and White Analytical Chemistry (GAC and WAC), with an Eco-Scale score of 87/100, AGREE 0.69, BAGI 80.0, RGB 95.4%, AGSA 91.67%, CaFRI 86%, and a favorable GAPI profile. Collectively, this study establishes CE-PDA as a sensitive, highly sustainable, and environmentally friendly analytical platform for investigating clinically important anticancer–antibiotic combinations.

## Data Availability

All data generated or analyzed during this study are included in this published article. Any additional datasets are available from the corresponding author upon reasonable request.
